# Association of Birth Asphyxia With Regional White Matter Abnormalities Among Patients With Schizophrenia and Bipolar Disorders

**DOI:** 10.1001/jamanetworkopen.2021.39759

**Published:** 2021-12-20

**Authors:** Laura A. Wortinger, Claudia Barth, Stener Nerland, Kjetil Nordbø Jørgensen, Alexey A. Shadrin, Attila Szabo, Unn Kristin Haukvik, Lars T. Westlye, Ole A. Andreassen, Marianne Thoresen, Ingrid Agartz

**Affiliations:** 1 Department of Psychiatric Research, Diakonhjemmet Hospital, Oslo, Norway; 2 Norwegian Centre for Mental Disorders Research, Institute of Clinical Medicine, University of Oslo, Oslo, Norway; 3 Norwegian Centre for Mental Disorders Research, Division of Mental Health and Addiction, Oslo University Hospital, Oslo, Norway; 4 Department of Medical Genetics, Oslo University Hospital, Oslo, Norway; 5 Centre of Research and Education in Forensic Psychiatry, Oslo University Hospital, Oslo, Norway; 6 Department of Adult Psychiatry, Institute of Clinical Medicine, University of Oslo, Oslo, Norway; 7 Department of Psychology, University of Oslo, Oslo, Norway; 8 KG Jebsen Centre for Neurodevelopmental Disorders, University of Oslo, Oslo, Norway; 9 Department of Physiology, Institute of Basic Medical Sciences, University of Oslo, Oslo, Norway; 10 Neonatal Neuroscience, Translational Health Sciences, University of Bristol, Bristol, United Kingdom; 11 Centre for Psychiatric Research, Department of Clinical Neuroscience, Karolinska Institutet, Stockholm, Sweden

## Abstract

**Importance:**

White matter (WM) abnormalities are commonly reported in psychiatric disorders. Whether peripartum insufficiencies in brain oxygenation, known as birth asphyxia, are associated with WM of patients with severe mental disorders is unclear.

**Objective:**

To examine the association between birth asphyxia and WM in adult patients with schizophrenia and bipolar disorders (BDs) compared with healthy adults.

**Design, Setting, and Participants:**

In this case-control study, all individuals participating in the ongoing Thematically Organized Psychosis project were linked to the Medical Birth Registry of Norway (MBRN), where a subset of 271 patients (case group) and 529 healthy individuals (control group) had undergone diffusion-weighted imaging (DWI). Statistical analyses were performed from June 16, 2020, to March 9, 2021.

**Exposures:**

Birth asphyxia was defined based on measures from standardized reporting at birth in the MBRN.

**Main Outcomes and Measures:**

Associations between birth asphyxia and WM regions of interest diffusion metrics, ie, fractional anisotropy (FA), axial diffusivity (AD), and radial diffusivity (RD), were compared between groups using analysis of covariance, adjusted for age, age squared, and sex.

**Results:**

Of the 850 adults included in the study, 271 were in the case group (140 [52%] female individuals; mean [SD] age, 28.64 [7.43] years) and 579 were in the control group (245 [42%] female individuals; mean [SD] age, 33.54 [8.31] years). Birth asphyxia measures were identified in 15% to 16% of participants, independent of group. The posterior limb of the internal capsule (PLIC) showed a significant diagnostic group × birth asphyxia interaction (*F*_(1, 843)_ = 11.46; *P* = .001), reflecting a stronger association between birth asphyxia and FA in the case group than the control group. RD, but not AD, also displayed a significant diagnostic group × birth asphyxia interaction (*F*_(1, 843)_ = 9.28; *P* = .002) in the PLIC, with higher values in patients with birth asphyxia and similar effect sizes as observed for FA.

**Conclusions and Relevance:**

In this case-control study, abnormalities in the PLIC of adult patients with birth asphyxia may suggest a greater susceptibility to hypoxia in patients with severe mental illness, which could lead to myelin damage or impeded brain development. Echoing recent early-stage schizophrenia studies, abnormalities of the PLIC are relevant to psychiatric disorders, as the PLIC contains important WM brain pathways associated with language, cognitive function, and sensory function, which are impaired in schizophrenia and BDs.

## Key Points

**Question:**

Are signs of insufficient brain oxygenation before, during, and immediately after birth, known as birth asphyxia, associated with white matter abnormalities in adult patients with schizophrenia and bipolar disorder compared with healthy control participants?

**Findings:**

In this case-control study of 850 adult patients and healthy individuals with birth registry and diffusion-weighted imaging data, measures of birth asphyxia were significantly associated with lower fractional anisotropy and higher radial diffusivity in the posterior limb of the internal capsule in the patients with bipolar disorder and schizophrenia but not in the control group.

**Meaning:**

White matter abnormalities in the posterior limb of the internal capsule could suggest early changes or impeded brain development because of an increased susceptibility to birth asphyxia in patients, which is not present in healthy adults.

## Teaser

This case-control study examines the association between birth asphyxia and white matter abnormalities in adult patients with schizophrenia and bipolar disorders.

## Introduction

Widespread white matter (WM) abnormalities in schizophrenia and bipolar disorder (BD) support the dysconnectivity hypothesis, which involves dysfunctional communication between brain regions^1,2,3,4^ and disturbances in WM microstructural organization.^5,6^ Furthermore, oligodendrocyte and myelin abnormalities in patients with schizophrenia and BD are commonly reported.^7,8,9^ Since premyelinating oligodendrocytes are key cellular targets in hypoxic brain injury,^10,11^ previous findings of WM abnormalities in patients might be partly accounted for by having experienced birth asphyxia.

Birth asphyxia is a condition in which affected newborns experience a peripartum deficiency in brain oxygenation. This can cause hypoxic injury and, in severe cases, lead to permanent brain damage or death in offspring. Common causes of birth asphyxia are mechanically obstructed labor (shoulder dystocia), abnormal fetal positioning, and placental abruption as well as dysfunction or maternal hypotension.^12^ A history of severe birth asphyxia often leads to cerebral palsy with or without cognitive deficit and has been associated with increased risk of developing schizophrenia (odds ratio, 4.4)^13^ and BD (hazard ratio, 5.3),^14^ but so have other serious obstetric complications (OCs).^15^

The genetic risk of developing schizophrenia is multiplied by a factor of 5 in the context of severe OCs,^16^ suggesting that a genetic liability for schizophrenia predisposes the developing fetus and newborn to these complications. However, severe OCs are defined by a broad range of perinatal complications (eg, birth asphyxia, discolored placenta/amniotic fluid, preeclampsia, gestational diabetes, birth ≤35 weeks, low birth weight ≤2000g). In our clinical cohort studies,^17^ we found significantly more cases of several severe OCs co-occurring in the presence of birth asphyxia (>50%) than when birth asphyxia was not present (<3%), confirming that birth asphyxia is more frequent in complicated pregnancies.

The Medical Birth Registry of Norway (MBRN) offers unique insight into the occurrence of birth asphyxia in the peripartum period, but it does not specify its severity (mild, moderate, or severe), its treatment, or information from neonatal magnetic resonance imaging (MRI) examinations. Also, inflammatory presentation increases vulnerability,^18^ so milder insults may cause injury or alterations in those with a genetic liability for schizophrenia development.^16^ Birth asphyxia had a prevalence of 14% in our previous studies, without case-control differences,^17,19,20^ suggesting that the birth asphyxia variable from the MBRN contains a wide range of birth asphyxia exposure.

Diffusion tensor imaging (DTI) is a method for indirectly characterizing WM microstructure and gross architecture. In DTI studies of individuals with schizophrenia and BD, WM abnormalities are commonly reported, with significantly lower fractional anisotropy (FA) in patients compared with healthy control participants.^5,6^ FA reflects the degree of diffusion directionality, and higher FA has often been interpreted as indicating better integrity.^21^ While FA is sensitive to structural alterations, the biological specificity is relatively low and cannot distinguish between different pathophysiological processes, such as dysmyelination or demyelination, inflammation, and axonal injury. Combining FA with axial diffusivity (AD) and radial diffusivity (RD), which reflect the diffusion along and perpendicular to the primary axis of the diffusion tensor, respectively, may allow for higher neurobiological specificity. For instance, decreased AD has been linked to axonal damage, while increased RD has been linked to either dysmyelination or demyelination.^21,22,23,24^

Using prospective birth registry and DTI data, the aims of this study were to (1) examine the prevalence of birth asphyxia measures in patients and healthy control participants; (2) test whether the association of a history of birth asphyxia with DTI metrics differed between patients and healthy control participants; (3) determine whether birth asphyxia–associated WM differences in patients are also associated with age of disease onset, disease severity, medication use, and symptoms.

## Methods

### Participants

This study is part of the Thematically Organized Psychosis (TOP) study, which is the main study protocol at the Norwegian Center for Mental Disorders Research (Oslo, Norway). All participants gave written informed consent, and the study was conducted in accordance with the Declaration of Helsinki^25^ and received approval from the Regional Committee for Medical Research Ethics–South East Norway. The Norwegian Data Inspectorate also approved the study. Patient and healthy control participant inclusion and exclusion criteria for the TOP study and sample information are described in the eMethods in the Supplement. This study followed the Strengthening the Reporting of Observational Studies in Epidemiology (STROBE) reporting guideline for case-control studies.

### Birth Asphyxia

Birth data were collected from the MBRN.^26^ Clinical features of severe birth asphyxia include neonatal encephalopathy (lethargy, stupor, or coma), an Apgar score of 5 or less at 10 minutes after birth, continued need for resuscitation, fetal acidosis, and others.^27^ While some information is not available in the MBRN (eg, lethargy, stupor, Apgar scores at 10 minutes, or fetal acidosis), birth asphyxia together with respiratory complications in affected newborns could indicate conditions contributing to insufficiencies in the supply of oxygen reaching the brain. Birth asphyxia was defined as having had a complication coded KP15 (threatening intrauterine asphyxia), AS54 (asphyxia and poor fetal sound), AS61 (asphyxia), 7762 (respiratory distress), 7763 (primary apnea), 7765 (atelectasis pulmonary), 7767 (suspended animation, irregular/slow heart reaction), 7768 (cyanosis attack), or 7769 (other hypoxic conditions/asphyxia) in the MBRN.

### Diffusion-Weighted Imaging Acquisition and Processing

We obtained diffusion-weighted imaging (DWI) from two 3T MRI scanners (Signa HDxt and Discovery MR750; GE) (eMethods in the Supplement). Preprocessing, including eddy current correction, echo-planar imaging–induced distortion correction, and tensor fitting, was performed as previously described.^28^ FA, AD, and RD maps were processed using tract-based spatial statistics,^29^ part of the FSL software.^30^ We extracted FA, AD, and RD from 34 lateral regions of interest (ROIs) representing both projection and association fibers and 5 ROIs from commissural fibers based on the intersection between the WM skeleton and relevant ROIs in probabilistic Johns Hopkins University WM atlases.^31,32^

To account for the effect of scanner and image acquisition protocols, we used ComBat to remove unwanted variation associated with scanner.^33,34^ ComBat harmonization was performed on the 34 lateral, 5 commissural, and average skeleton values for FA and diffusivity measures separately (eFigures 1 to 3 in the Supplement). Age, sex, diagnostic group, and birth asphyxia were entered as variables of interest.

After ComBat harmonization, 17 bilateral WM ROIs (averaged from the 34 unilateral ROIs), 2 bilateral ROIs (averaged from 6 unilateral ROIs each), 5 commissural ROIs, and average skeleton FA, AD, or RD resulted in a total of 25 ROIs, representing all major WM fasciculi,^6^ and were used in the main analysis. Since the 2 hemispheres may show differential associations with birth asphyxia, we examined significant regions in each hemisphere separately in follow-up analyses.

### Clinical Variables

From the structured clinical interview, information was obtained about the age of disease onset, disease severity (using the Global Assessment of Functioning scale [GAF]),^35^ medication use and psychotic symptoms (using the Positive and Negative Syndrome Scale [PANSS]).^36^ Age of disease onset was assessed retrospectively and defined as the year of the first occurrence of psychotic symptoms (equivalent to having had 4 or more on selected PANSS items for a week or longer) for patients with psychotic disorders and the year of first occurrence of a major mood episode (depressive, hypomanic, manic, or mixed episode) for patients with BDs. Current medication use was assessed by clinical interviews and/or medical record review.

### Statistical Analysis

#### Clinical, Demographic, and Birth Asphyxia Variables

Within and between-group comparisons were performed with 1-way analysis of variance for continuous variables and Pearson χ^2^ tests for categorical variables. Post hoc, pairwise group comparisons were performed if there were significant main effect sizes of group. Statistical analyses were performed using SPSS version 27 (IBM Corp).

#### WM ROIs and Birth Asphyxia Between Groups

We assessed the 25 WM FA ROIs using a full factorial 2 × 2 analysis of covariance with birth asphyxia (birth asphyxia positive or negative) and group (case or control) included as between-group factors, covarying for age, age squared, and sex. Age squared was added to model the association of age more accurately, given that both linear and nonlinear age effects have been reported for FA.^37,38^ For our primary FA analysis of whether the associations of birth asphyxia differed between individuals in the case and control groups, results of interaction tests were declared significant if they survived correction for multiple comparisons, with a Bonferroni correction threshold of 0.05 / 25, or *P* = .002. Effect sizes were reported as Cohen *d* values^38^ for birth asphyxia–positive/birth asphyxia–negative differences within groups.

For significant FA regions from the main analysis, follow-up analyses were performed to examine associations in diagnostic subgroups, RD, and AD. Because we were interested in neural abnormalities associated with birth asphyxia and their further association with clinical outcomes, follow-up analyses were performed to explore whether variation in FA and its interactions with group and birth asphyxia were associated with age of disease onset, chlorpromazine equivalence scores, PANSS scores, or GAF scores in separate multivariable linear regression models. These analyses are described in detail in the eMethods in the Supplement. Given the well-established dependence of FA on age,^37,38^ sensitivity analyses were performed within both case and control groups separately to assess whether differences in birth asphyxia were still present.

## Results

### Clinical and Demographic Variables

Of the 850 adults included in the study, 271 were in the case group (140 [52%] female individuals; mean [SD] age, 28.64 [7.43] years) and 579 were in the control group (245 [42%] female individuals; mean [SD] age, 33.54 [8.31] years). In the case group, 111 (41%) had BD, and 160 (59%) had schizophrenia. Clinical and demographic data are presented in Table 1. There were no significant differences between the control, BD, and schizophrenia groups in birth weight (mean [SD]: control, 3506.53 [583.49] g; BD, 3608.81 [586.29] g; schizophrenia, 3460.04 [582.76] g), gestational age (mean [SD]: control, 39.78 [1.89] weeks; BD, 39.83 [1.75] weeks; schizophrenia, 39.47 [2.50] weeks), birth head circumference (mean [SD]: control, 35.20 [1.46] cm; BD, 35.40 [1.46] cm; schizophrenia, 35.12 [1.45] cm), or birth length (mean [SD]: control, 50.21 [2.36] cm; BD, 50.65 [2.36] cm; schizophrenia, 50.04 [2.42] cm). In patients with BD compared with those with schizophrenia, significantly greater birth weight (mean [SD]: BD, 3608.81 [586.29] g; schizophrenia, 3460.04 [582.76] g) and longer birth length (mean [SD]: BD, 50.65 [2.36] cm; SZ, 50.04 [2.42] cm) were found.

**Table 1.** Clinical and Demographic Variables

Characteristic	CG			BDG			SG			Main effect size		Pairwise					
	No BA (n = 486)	BA (n = 93)	F (*df*)	No BA (n = 94)	BA (n = 17)	F (*df*)	No BA (n = 134)	BA (n = 26)	F (*df*)	F or χ^2^ (*df*)	*P* value	SG vs CG, difference, mean (SEM)	*P* value	BD vs CG, difference, mean (SEM)	*P* value	SG vs BDG, difference, mean (SEM)	*P* value
Sex, No. (%)																	
Female	214 (44)	31 (33)	3.66 (1, 579)^a^^,^^b^	55 (59)	12 (71)	0.89 (1, 111)^b^	59 (44)	14 (54)	0.85 (1, 160)^b^	12.25 (2)^b^	.002	NA	.45	BD>HC	<.001	BD>SZ	.02
Male	272 (56)	62 (67)		39 (42)	5 (29)		75 (56)	12 (46)									
NR, No. (%)^c^	61 (13)	7 (8)	1.92 (1, 566)^b^	7 (8)	3 (20)	2.18 (1, 104)^b^	11 (9)	4 (17)	1.61 (1, 149)^b^	0.80 (2)^b^	.67	NA	.44	NA	.48	NA	.91
Age, mean (SD), y	33.83 (8.43)	32.01 (7.49)	3.77 (1, 577)	28.88 (7.72)	28.76 (6.98)	0.00 (1, 109)	28.34 (7.23)	29.23 (8.01)	0.32 (1, 158)	34.29 (2, 847)	<.001	−5.05 (0.72)	<.001	−4.67 (0.83)	<.001	−0.38 (0.99)	.70
Education, mean (SD), y^d^	14.72 (2.13)	14.43 (2.13)	1.41 (1, 565)	13.72 (2.27)	13.53 (1.51)	0.09 (1, 101)	12.50 (1.95)	12.12 (2.03)	0.80 (1, 152)	70.41 (2, 824)	<.001	−2.23 (0.19)	<.001	−0.98 (0.23)	<.001	−1.25 (0.27)	<.001
Adult height, mean (SD), cm^e^^,^^f^	176.66 (5.95)	175.44 (5.86)	2.48 (1, 392)	174.28 (6.23)	172.76 (6.27)	0.85 (1, 106)	175.10 (7.04)	173.22 (7.09)	1.54 (1, 152)	1.41 (2, 655)	.25	−0.69 (0.59)	.25	0.60 (0.68)	.38	−1.29 (0.78)	.10
Birth measures																	
BW, mean (SD), g^e^^,^^g^	3524 (573.18)	3438 (578.62)	1.74 (1, 576)	3612 (484.77)	3471 (482.40)	1.21 (1, 108)	3500 (648.25)	3254 (652.67)	3.09 (1, 157)	2.17 (2, 846)	.12	−46.50 (52.07)	.37	102.28 (60.82)	.10	−148.77 (72.23)	.04
GA, mean (SD), wk^h^	39.76 (1.76)	39.86 (2.47)	0.20 (1, 549)	39.80 (1.76)	40.00 (1.75)	0.17 (1, 100)	39.57 (2.03)	39.00 (4.15)	1.08 (1, 150)	1.56 (2, 802)	.21	−0.31 (0.18)	.10	0.05 (0.22)	.81	−0.36 (0.26)	.16
BHC, mean (SD), cm^e^^,^^i^	35.23 (1.54)	35.14 (1.47)	0.20 (1, 357)	35.36 (1.42)	35.21 (1.42)	0.12 (1, 90)	35.11 (1.39)	35.31 (1.44)	0.28 (1, 128)	0.99 (2, 580)	.37	−0.08 (0.15)	.60	0.19 (0.17)	.19	−0.27 (0.20)	.17
BL, mean (SD), cm^e^^,^^j^	50.26 (2.41)	50.12 (2.36)	0.24 (1, 565)	50.52 (1.92)	50.63 (1.90)	0.05 (1, 106)	50.16 (2.69)	49.32 (2.67)	1.85 (1, 145)	2.23 (2, 821)	.11	−0.17 (0.22)	.44	0.44 (0.25)	.07	−0.60 (0.30)	.04
Age at onset, mean (SD), y	NA	NA	NA	18.21 (5.22)	16.77 (3.95)	1.18 (1, 109)	21.70 (6.22)	21.92 (5.54)	0.03 (1, 155)	28.20 (1, 266)	<.001	NA	NA	NA	NA	3.75 (0.71)	<.001
Scale scores, mean (SD)																	
GAF-S	NA	NA	NA	62.02 (9.31)	61.35 (8.85)	0.08 (1, 109)	47.78 (13.77)	52.04 (14.84)	2.03 (1, 158)	78.77 (1, 269)	<.001	NA	NA	NA	NA	−13.44 (1.52)	<.001
GAF-F	NA	NA	NA	61.37 (10.92)	62.82 (10.92)	0.26 (1, 108)	49.04 (13.67)	52.00 (14.78)	1.00 (1, 158)	58.66 (1, 268)	<.001	NA	NA	NA	NA	−12.07 (1.58)	<.001
PANSS	NA	NA	NA	42.45 (7.73)	44.29 (5.72)	0.88 (1, 108)	59.84 (16.44)	54.81 (10.68)	2.25 (1, 158)	102.03 (1, 268)	<.001	NA	NA	NA	NA	16.29 (1.61)	<.001
PANSS general	NA	NA	NA	24.19 (4.41)	25.41 (3.81)	1.14 (1, 109)	30.89 (8.10)	29.00 (5.15)	1.31 (1, 158)	58.77 (1, 269)	<.001	NA	NA	NA	NA	6.20 (0.81)	<.001
PANSS negative	NA	NA	NA	9.55 (2.99)	9.88 (2.98)	0.18 (1, 108)	15.14 (5.91)	13.08 (4.43)	2.86 (1, 158)	76.50 (1, 268)	<.001	NA	NA	NA	NA	5.21 (0.60)	<.001
PANSS positive	NA	NA	NA	8.68 (2.05)	9.00 (2.29)	0.34 (1, 109)	13.81 (4.88)	12.73 (4.09)	1.13 (1, 158)	103.90 (1, 269)	<.001	NA	NA	NA	NA	4.91 (0.48)	<.001
APD																	
CPZ equivalent APD dose, mean (SD)	NA	NA	NA	182.29 (122.08)	271.78 (159.69)	2.85 (1, 41)	299.27 (207.30)	256.35 (142.50)	0.86 (1, 129)	8.67 (1, 172)	.004	NA	NA	NA	NA	95.21 (32.34)	.004
Taking APD, No. (%)	NA	NA	NA	36 (38)	7 (41)	0.05 (1 111)^b^	112 (84)	22 (85)	0.02 (1, 160)^b^	NA	NA	NA	NA	NA	NA	(SZ>BD)	<.001
Taking lithium, No. (%)	NA	NA	NA	16 (17)	2 (12)	0.29 (1, 111)^b^	4 (3)	3 (12)	3.81 (1, 160)^b^	NA	NA	NA	NA	NA	NA	(BD>SZ)	<.001
Psychotic symptoms, No. (%)	NA	NA	NA	51 (54)	9 (53)	0.01 (1, 111)^b^	NA	NA	NA	NA	NA	NA	NA	NA	NA	NA	NA

Abbreviations: APD, antipsychotic drug; BA, birth asphyxia; BD, bipolar disorder spectrum group; BHC, birth head circumference; BL, birth length; BW, birth weight; CG, control group; CPZ, chlorpromazine; *df*, degrees of freedom; GA, gestational age; GAF-S, Global Assessment of Functioning–Symptoms; GAF-F, Global Assessment of Functioning–Functioning; NR, non–right handed; PANSS, Positive and Negative Syndrome Scale; SG, schizophrenia spectrum group.

^a^ *P* = .04, per 1-tailed Fisher exact test.

^b^ Values are χ^2^ with *df*.

^c^ Total sample size included 475 patients without birth asphyxia and 91 with in the control group; 89 without birth asphyxia and 15 with in the BD group, and 126 patients without birth asphyxia and 23 with in the schizophrenia group.

^d^ Total sample size included 476 patients without birth asphyxia and 91 with in the control group; 88 without birth asphyxia and 15 with in the BD group, and 129 patients without birth asphyxia and 25 with in the schizophrenia group.

^e^ Means are adjusted for sex.

^f^ Total sample size included 325 patients without birth asphyxia and 70 with in the control group; 92 without birth asphyxia and 17 with in the BD group, and 129 patients without birth asphyxia and 26 with in the schizophrenia group.

^g^ Total sample size included 486 patients without birth asphyxia and 93 with in the control group; 94 without birth asphyxia and 17 with in the BD group, and 134 patients without birth asphyxia and 26 with in the schizophrenia group.

^h^ Total sample size included 463 patients without birth asphyxia and 88 with in the control group; 86 without birth asphyxia and 16 with in the BD group, and 127 patients without birth asphyxia and 25 with in the schizophrenia group.

^i^ Total sample size included 293 patients without birth asphyxia and 67 with in the control group; 79 without birth asphyxia and 14 with in the BD group, and 114 patients without birth asphyxia and 17 with in the schizophrenia group.

^j^ Total sample size included 479 patients without birth asphyxia and 89 with in the control group; 92 without birth asphyxia and 17 with in the BD group, and 126 patients without birth asphyxia and 22 with in the schizophrenia group.

### Birth Asphyxia

Birth asphyxia was identified in 93 of 579 participants in the control group (16%), 17 of 111 in the BD group (15%), and 26 of 160 in the schizophrenia group (16%), and this prevalence did not significantly differ between groups (Table 2). Of those with birth asphyxia in the control group, significantly more male participants experienced birth asphyxia than female participants (62 [67%] vs 31 [33%]; *P* = .04).^16^ There were no significant sex differences in those who experienced birth asphyxia in the BD and schizophrenia groups.

**Table 2.** Frequency of Birth Asphyxia

Outcome	CG (n = 579)			BDG (n = 111)			SG (n = 160)			SG vs CG		BDG vs CG		SG vs BD	
	No BA, No.	BA, No.	Total No. (%)^a^	No BA, No.	BA, No.	Total No. (%)^a^	No BA, No.	BA, No.	Total No. (%)^a^	χ^2^	*P* value	χ^2^	*P* value	χ^2^	*P* value
Birth asphyxia	0	93	93 (16.1)	0	17	17 (15.3)	0	26	26 (16.3)	0.00	.95	0.04	.84	0.04	.84
Asphyxia/hypoxia	0	81	81 (14.0)	0	17	17 (15.3)	0	19	19 (11.9)	0.48	.49	0.13	.71	0.67	.41
Respiratory complications															
Distress, apnea, atelectasis pulmonary, almost dead, cyanosis	0	2	2 (0.3)	0	0	0	1	4	5 (3.1)	10.32	.001	0.39	.54	3.53	.06
Threatening intrauterine asphyxia	12	4	16 (2.8)	0	2	2 (1.8)	6	3	9 (5.6)	3.14	.08	0.34	.56	2.46	.12
Preterm birth (≤35 wk)	7	6	13 (2.2)	2	1	3 (2.7)	7	4	11 (6.9)	8.55	.003	0.09	.77	2.33	.13
Low birth weight (≤2500 g)	14	10	24 (4.1)	0	0	0	5	5	10 (6.3)	1.27	.26	4.77	.03	7.2	.007
Apgar score 0-7 at 1 or 5 min	9	21	30 (5.2)	4	7	11 (9.9)	9	12	21 (13.1)	12.31	<.001	3.73	.05	0.65	.42
Apgar score 0-3 at 1 min or 0-7 at 5 min	1	6	7 (1.2)	0	4	4 (3.6)	1	2	3 (1.9)	0.42	.52	3.41	.07	0.78	.38
Preeclampsia	11	2	13 (2.2)	3	0	3 (2.7)	0	0	0	3.66	.06	0.09	.77	4.37	.04
Gestational diabetes	1	0	1 (0.2)	1	0	1 (0.9)	1	0	1 (0.6)	0.95	.33	1.71	.19	0.07	.79
Umbilical cord prolapse	1	3	4 (0.7)	0	0	0	0	0	0	1.11	.29	0.77	.38	NA	NA
Placental abruption	0	0	0)	0	0	0	0	1	1	3.62	.06	NA	NA	0.70	.40
Breech delivery	14	7	21 (3.6)	5	1	6 (5.4)	11	2	13 (8.1)	5.78	.02	0.78	.38	0.74	.39
Emergency cesarean delivery	7	7	14 (2.4)	4	1	5 (4.5)	5	4	9 (5.6)	4.28	.04	1.51	.22	0.17	.68
Shoulder dystocia	2	3	5 (0.9)	1	0	1 (0.9)	0	1	1 (0.6)	0.09	.77	0.00	.97	0.07	.79
Discolored placenta or amniotic fluid	30	38	68 (11.7)	8	7	15 (13.5)	10	6	16 (10)	0.38	.54	0.28	.60	0.80	.37

Abbreviations: BA, birth asphyxia; BDG, bipolar spectrum group; CG, control group; SG, schizophrenia spectrum group.

^a^ Percentage represents the frequency of the condition within each diagnostic subgroup.

Comparing the schizophrenia group with the control group, there were significantly more cases of preterm birth (11 [7%] vs 13 [2%]), low Apgar scores (21 [13%] vs 30 [5%]), breech deliveries (13 [8%] vs 21 [4%]), and emergency cesarean deliveries (9 [6%] vs 14 [2%]). Additionally, we found significantly more severe OCs co-occurred in the presence of birth asphyxia (eg, 70 of 93 control group participants [75%]), than when birth asphyxia was not present (82 of 486 control group participants [17%]), across groups (Table 3).

**Table 3.** Co-occurring Obstetric Complications by Diagnostic Subgroup^a^

Diagnostic subgroup	Participants, No./total No.(%)	χ^2^	*P* value
Control group			
No BA	82/486 (17)	137.50	<.001
BA	70/93 (75)		
BD group			
No BA	21/94 (22)	26.02	<.001
BA	14/17 (82)		
Schizophrenia group			
No BA	29/134 (22)	27.43	<.001
BA	19/26 (73)		

Abbreviation: BA, birth asphyxia.

^a^ Co-occurring obstetric complications include 1 or more of the following: preterm birth, low birth weight, low Apgar score (0-3 at 1 minute or 0-7 at 5 minutes), preeclampsia, gestational diabetes, umbilical cord prolapse, placental abruption, breech delivery, emergency cesarean delivery, shoulder dystocia, or discolored placenta/amniotic fluid, with or without the presence of BA.

### WM ROIs and Birth Asphyxia Between Groups

For the 25 ROIs, no significant main associations of birth asphyxia were observed (eTable in the Supplement). A statistically significant interaction between birth asphyxia and diagnostic group was found for the posterior limb of the internal capsule (PLIC) (*F*_(1, 843)_ = 11.46; *P* = .001), which survived correction for multiple comparisons. The significant interaction revealed lower mean (SD) FA in patients with birth asphyxia (0.68 [0.02]) compared with patients without birth asphyxia (0.69 [0.02]), which was not found in the control group (mean [SD] among healthy participants with birth asphyxia, 0.69 [0.02]; without birth asphyxia, 0.68 [0.02]) (eTable in the Supplement).

In follow-up analyses on FA in the PLIC, a significant interaction between birth asphyxia and diagnostic subgroups (*F*_(2, 841)_ = 5.66; *P* = .004) revealed the lowest mean (SD) FA in patients with birth asphyxia and schizophrenia (with birth asphyxia, 0.68 [0.02]; without, 0.69 [0.02]; *d* = −0.46) followed by those with birth asphyxia and BD (with birth asphyxia, 0.68 [0.02]; without, 0.69 [0.02]; *d* = −0.40), with similar effect sizes in both groups. In the control group, higher mean (SD) FA was shown in the group with birth asphyxia (with birth asphyxia, 0.69 [0.02]; without, 0.68 [0.02]; *d* = 0.24) (Figure). This pattern was found in individual analyses of both the left and right PLIC-FA (eAppendix 1 and eFigure 4 in the Supplement).

**Figure.**
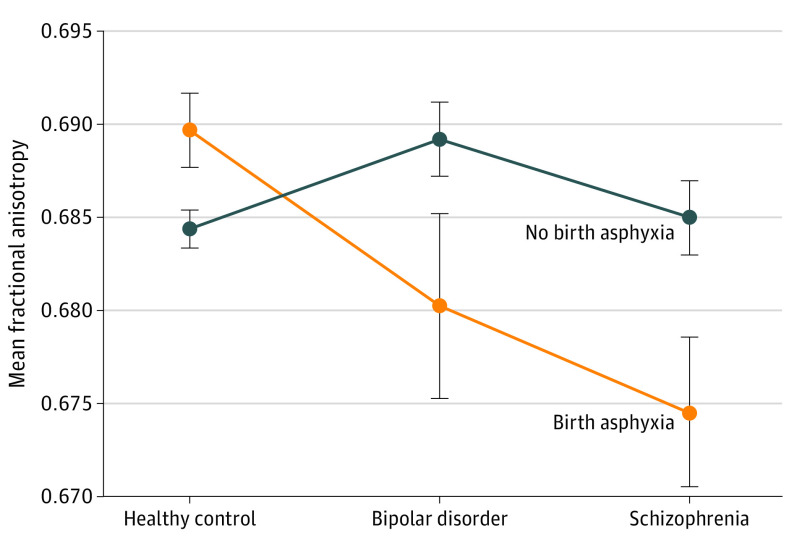
Diagnostic Subgroup × Birth Asphyxia Interaction for Fractional Anisotropy in the Posterior Limb of the Internal Capsule Error bars indicate standard error of the mean.

In follow-up analyses of the PLIC, a significant interaction between birth asphyxia and diagnostic group was observed for RD (*F*_(1, 843)_ = 9.28, *P* = .002) but not for AD (*F*_(2, 841)_ = 2.49, *P* = .12) and showed higher mean (SD) values in patients with birth asphyxia (0.37 [0.05]) compared with those without (0.36 [0.02]), which was not found in the control group (with birth asphyxia, 0.36 [0.05]; without, 0.36 [0.02]). Additional follow-up analyses are reported in eAppendix 1, eFigure 5, and eFigure 6 in the Supplement.

## Discussion

We found an equal prevalence of birth asphyxia between patients and healthy individuals in the control group. The association of birth asphyxia with DTI measures was specific to patients, with significantly lower FA in the PLIC of patients with birth asphyxia compared with patients without birth asphyxia. RD of the PLIC showed similar deviations as FA, with higher values in patients with birth asphyxia; effect sizes were also similar.

Together with the smaller basal ganglia and thalamic volumes demonstrated in a previous birth asphyxia study on an overlapping sample,^17^ the WM abnormalities in the PLIC of patients with birth asphyxia are consistent with MRI findings of asphyxiated infants with the poorest outcomes.^12,39,40^ The PLIC contains fibers of the superior thalamic radiation, corticospinal, corticofugal, and corticopontine tracts that support important motor, language, cognitive, and sensory functions,^40^ often found to be impaired in individuals with BD and schizophrenia.^41,42,43,44^ Even though participants with neurological disorders or neuroradiological MRI findings indicating brain pathology were excluded (eAppendix 2 in the Supplement), we were still able to find abnormalities in the PLIC of patients with birth asphyxia, which might suggest early damage or impeded brain development.

Interestingly, the PLIC was one of the few WM regions where no FA abnormalities were detected in adult patients with schizophrenia and BD in 2 large meta-analyses conducted by the ENIGMA SZ^6^ and BD^5^ working groups. In an early-onset schizophrenia study, lower FA of the PLIC was reported.^45^ The corticospinal tract, constituting a large part of the PLIC, showed the lowest FA in early-onset schizophrenia, but differences became less prominent over time.^46^ Application of machine learning in a structural and diffusion imaging study found one of most discriminative features of case-control status was FA of the corticospinal tract and PLIC in first-episode schizophrenia.^47^ In a 6-week trial of risperidone on patients with schizophrenia not previously receiving medication, increased orientation dispersion index (ODI), a biophysical characterization of WM microstructure, was only detected in the PLIC, and increases in whole brain ODI were associated with poorer responses to treatment.^48^ Even though differences within the PLIC were not evident in large meta-analysis data sets,^5,6^ it could be that delayed maturation of corticospinal tract, differences in disease stages, or differences in medication use could explain these discrepancies.

Genetic and environmental factors are thought to disrupt normal development from early in life, resulting in cumulative molecular and histogenic responses,^49,50,51^ which may not be fully captured in large data sets of chronic adult patients and healthy control participants. Considering the interacting environment of pregnancy and birth, the interplay between birth asphyxia and diagnosis may explain more of the variability in PLIC, which is not revealed with simple main effects. Given that birth asphyxia was equally occurring in all groups, our findings support the idea that having a history of birth asphyxia is an interactive, rather than a directly causal, risk factor in the development of a psychiatric disorder.

Severe hypoxia-ischemia in preterm or term infants tends to affect brain regions in the border zones between the end fields of major cerebral arteries (eg, parasagittal cortex and hippocampus) and in areas that are actively myelinating (eg, thalamus, basal ganglia, and PLIC).^10,11,52^ Higher RD values were significantly associated with the PLIC of patients with birth asphyxia, which gives an indication that the low FA values could be driven by abnormalities in myelin. Myelination of axons progresses from the brainstem and deep brain structures prior to birth to peripheral brain regions mainly during the first year after birth but also throughout life.^53,54,55^ Myelination of deep brain structures, like the ventro-lateral nuclei and sub-thalamic nucleus, begins first at 29 weeks’ gestational age and then in the PLIC at 39 to 40 weeks.^56^

Myelination of the PLIC is considered an important landmark for evaluating neonatal brain development.^57^ Because gestational age was similar between groups (39-40 weeks), it could be that PLIC abnormalities in patients with birth asphyxia may be associated with genetics or other mechanisms underlying both myelination and an intolerance to hypoxia, which was not found in the individuals in the control group. The functional hypoxia-inducible lipid droplet-associated (HILPDA) protein is a novel regulator of intracellular lipid and energy metabolism expressed under hypoxia.^58^ HILPDA expression, attenuated in schizophrenia,^59^ might represent a protective factor that explains the higher FA in the PLIC of individuals in the control group with birth asphyxia.

### Limitations

This study has limitations, including the wide definition and lack of details in the measures of birth asphyxia from the MBRN. The wide definition might account for the high prevalence (>15%) of birth asphyxia, as birth asphyxia resulting in hypoxic-ischemic encephalopathy only occurs in 1.8 per 1000 live births,^60^ and the prevalence reported here is nearly 100 times more. We found an equal prevalence of birth asphyxia among the groups, which is unexpected if genetic risk of schizophrenia predisposes patients to early insults around birth. Since obvious organic pathology and/or brain lesions in their MR images and neurological disorders precluded recruitment, it could be that the birth asphyxia measure and design of the TOP study do not fully capture the severe end of the hypoxic spectrum, which results in a series of functional deficits later in life. Even so, the birth asphyxia measures from the MBRN are likely associated with a range of hypoxia and asphyxia exposures, which were less tolerated because of genetic susceptibilities already present in newborns who later developed a psychiatric disorder.

We studied adults, whereas examining myelination trajectories in newborns and during early development would be of interest for future studies. Another limitation is the use of WM DTI metrics, which lack biological specificity. DTI metrics can be affected by various neurobiological processes, such as neuroinflammation, edema, and WM fiber crossing,^61^ which have been reported in patients with shizophrenia^62^ and BD.^63^ Future studies including myelin-sensitive MRI sequences, such as multishell diffusion MRI metrics,^51^ may allow for stronger biological interpretations.

## Conclusions

This study reported DTI abnormalities in the PLIC of patients with birth asphyxia that were not detected in the control group, potentially indicating early myelin damage or impeded brain development caused by an intolerance to birth asphyxia. Abnormalities of in the PLIC might be especially relevant to patients because the PLIC contains important WM brain pathways associated with language, cognitive functions, and sensory functions, which are impaired in schizophrenia and BD. Results should be replicated in independent samples.

## Notes

Supplement. **eMethods. **Participants, DWI Acquisition, and Statistical Analyses **eAppendix 1. **Supplementary Results **eTable. **Twenty-five FA ROIs **eFigure 1. **FA Comparison Before and After ComBat Harmonization for Scanners **eFigure 2.** RD Comparison Before and After ComBat Harmonization for Scanners **eFigure 3.** AD Comparison Before and After ComBat Harmonization for Scanners **eFigure 4.** Diagnostic Subgroup × Birth Asphyxia Interaction for Fractional Anisotropy in the Left and Right Hemisphere Posterior Limb of the Internal Capsule **eFigure 5. **Diagnostic Subgroup × Birth Asphyxia Interaction for Radial Diffusivity in the Posterior Limb of the Internal Capsule **eFigure 6. **Diagnostic Subgroup × Birth Asphyxia Interaction for Radial Diffusivity in the Left and Right Hemisphere Posterior Limb of the Internal Capsule **eAppendix 2.** TOP MRI Grading Scheme
eReferences.
