# Errors in Methods and Table

**DOI:** 10.1001/jamanetworkopen.2021.47966

**Published:** 2022-01-18

In the Original Investigation titled “Association of Birth Asphyxia With Regional White Matter Abnormalities Among Patients With Schizophrenia and Bipolar Disorders,”^1^ published December 20, 2021, there were errors in the Statistical Analysis subsection of the Methods section and in Table 1. In the first paragraph of the Statistical Analysis, the second sentence should have read that post hoc, pairwise group comparisons were performed if there were significant group associations. In Table 1, a heading was incorrectly labeled “Main effect size”; it should have read “Group associations.” This article has been corrected.^1^
