# Supplementary material for: Association of Birth Asphyxia With Regional White Matter Abnormalities Among Patients With Schizophrenia and Bipolar Disorders
Source: JAMA Netw Open. 2021 Dec 20;4(12):e2139759. doi: 10.1001/jamanetworkopen.2021.39759 (PMC8689382; doi:10.1001/jamanetworkopen.2021.39759)
Supplement: Supplement. — eMethods. Participants, DWI Acquisition, and Statistical Analyses eAppendix 1. Supplementary Results eTable. Twenty-five FA ROIs eFigure 1. FA Comparison Before and After ComBat Harmonization for Scanners eFigure 2. RD Comparison Before and After ComBat Harmonization for Scanners eFigure 3. AD Comparison Before and After ComBat Harmonization for Scanners eFigure 4. Diagnostic Subgroup × Birth Asphyxia Interaction for Fractional Anisotropy in the Left and Right Hemisphere Posterior Limb of the Internal Capsule eFigure 5. Diagnostic Subgroup × Birth Asphyxia Interaction for Radial Diffusivity in the Posterior Limb of the Internal Capsule eFigure 6. Diagnostic Subgroup × Birth Asphyxia Interaction for Radial Diffusivity in the Left and Right Hemisphere Posterior Limb of the Internal Capsule eAppendix 2. TOP MRI Grading Scheme eReferences. [file jamanetwopen-e2139759-s001.pdf]

Supplemental Online Content

Wortinger LA, Barth C, Nerland S, et al. Association of birth asphyxia with regional white matter abnormalities among patients with schizophrenia and bipolar disorders. *JAMA Netw Open.* 2021;4(12):e2139759. doi:10.1001/jamanetworkopen.2021.39759

eMethods. Participants, DWI Acquisition, and Statistical Analyses

eAppendix 1. Supplementary Results

eTable. Twenty-five FA ROIs

eFigure 1. FA Comparison Before and After ComBat Harmonization for Scanners

eFigure 2. RD Comparison Before and After ComBat Harmonization for Scanners

eFigure 3. AD Comparison Before and After ComBat Harmonization for Scanners

eFigure 4. Diagnostic Subgroup × Birth Asphyxia Interaction for Fractional Anisotropy in the Left and Right Hemisphere Posterior Limb of the Internal Capsule

eFigure 5. Diagnostic Subgroup × Birth Asphyxia Interaction for Radial Diffusivity in the Posterior Limb of the Internal Capsule

eFigure 6. Diagnostic Subgroup × Birth Asphyxia Interaction for Radial Diffusivity in the Left and Right Hemisphere Posterior Limb of the Internal Capsule

eAppendix 2. TOP MRI Grading Scheme

eReferences.

This supplemental material has been provided by the authors to give readers additional information about their work.

© 2021 Wortinger LA et al. *JAMA Network Open.*

eMethods. Participants, DWI Acquisition, and Statistical Analyses

Participants

Since October 2002, adult patients on the schizophrenia and bipolar disorder spectrums have been recruited consecutively from psychiatric units (outpatient and inpatient) of public hospitals in the Oslo region. The healthy controls (HC) were randomly selected from the national population register in the same catchment area as the patients. All patients underwent thorough clinical investigation by trained psychologists and physicians. Clinical diagnoses were assessed using the Structured Clinical Interview for DSM-IV axis 1 disorder (SCID-I) module A-E [1]. Psychosocial function was assessed with the Global Assessment of Functioning scale, split version (GAF) [2]. Current psychotic symptoms were rated by the use of the Positive and Negative Syndrome Scale (PANSS) [3]. HC were interviewed by trained research assistants and examined with the Primary Care Evaluation of Mental Disorders (Prime-MD) to ensure no current or previous psychiatric disorders [4].

Exclusion criteria for both patients and HC were organic disorders (pure drug-induced condition, somatic health condition, brain damage or head trauma with unconsciousness over 5 min, neurological diseases, autism spectrum disorder) and age outside the range of 18–65 years. All MRI scans were assessed by a neuroradiologist using a graded scheme (See TOP MRI Grading Scheme below or [5,6] for similar grading schemes), and if pathology findings were detected, the participant was excluded. Additional exclusion criteria for HC were current or previous somatic illness and substance misuse disorders or dependency within the last 6 months. HC were also excluded if they or a first-degree relative had a lifetime history of severe psychiatric disorder.

The sample (N = 850) consisted of patients with a DSM-IV diagnosis within the *schizophrenia spectrum* (SZ): schizophrenia (DSM-IV 295.1, 295.3, 295.6, and 295.9; n = 80), schizophreniform disorder (DSM-IV 295.4; n = 10), schizoaffective disorder (DSM-IV 295.7; n = 25), other psychosis (psychosis not otherwise specified, DSM-IV 298.9, n = 32; brief psychotic disorder, DSM-IV 298.8, n = 5; delusional disorder, DSM-IV 297.1, n = 8); or within the *bipolar disorder spectrum* (BD): Bipolar I disorder (DSM-IV 296.0–7; n = 57), Bipolar II disorder (DSM-IV 296.89; n = 49) or bipolar disorder not otherwise specified (DSM-IV 296.80; n = 5); and HC (n = 579).

DWI acquisition

Imaging was performed on two 3T MRI scanners at the Oslo University Hospital, the General Electric Signa HDxt with an 8-channel head coil and General Electric Discovery MR750 with a 32-channel head coil. Patients and healthy controls were scanned interchangeably/continuously between 2012 and 2018 (median year: 2015), which reduces the risk of potential bias in case/control comparisons due to long term scanner drift or minor upgrades occurring during the study period.

381 participants (278 HC, 34 BD and 69 SZ patients) underwent imaging on the General Electric Signa HDxt. DWI was acquired with a spin-echo planar imaging (EPI) sequence with the following parameters: TR/TE/flip angle: 15000 ms/85 ms/ 90°, slice thickness: 2.5 mm; in-plane resolution: 1.9 mm. We obtained 30 volumes of diffusion weighted data with different gradient directions (b = 1000 s/mm^2), and two b = 0 volumes with reversed phase-encoding direction (blip up/down) were acquired for correction of susceptibility distortions.

469 participants (301 HC, 77 BD and 91 SZ patients) underwent imaging on the General Electric Discovery MR750. DWI was acquired with an EPI sequence with the following parameters: TR/TE/flip angle: 8150 ms/83 ms/90°, slice thickness: 2 mm, in-plane resolution: 2 mm. We obtained 10 volumes of $b = 0$ and diffusion weighted data along 60 (b = 1000s/mm^2) and 30 (b = 2000s/mm^2) diffusion weighted volumes. In addition, 7 b = 0 volumes with reversed phase-encoding direction were acquired for correction of susceptibility distortions.

Statistical analyses

In follow-up analyses on significant results, we performed a 2 x 3 ANCOVA with ASPH (ASPH+/ASPH−) and diagnostic subgroups (BD, SZ and HC) included as between-group factors, covarying for age, age^2 and sex. We also assessed significant ROIs in the left and right hemisphere separately using the same analysis model. On significant FA ROIs, RD and AD analyses were performed using the same model as well. RD values were small, so we multiplied group means by 1000 for easier presentation.

© 2021 Wortinger LA et al. *JAMA Network Open.*

To assess the effects of diagnostic subgroup and significant FA regions on clinical outcome variables (age of disease onset, CPZ scores, PANSS or GAF) in separate multiple regression analyses, SZ was dummy coded with BD as a reference, and entered, together with FA and ASPH, as independent variables. The FA and SZ interaction term were added as FA-by-SZ. We used an interaction term with ASPH to determine whether ASPH moderated the relationship between FA (ASPH-by-FA) and the outcome variables. Also, interaction terms with ASPH were used to determine whether ASPH interacted with the variable, diagnostic subgroup, that was driven by the contrast (SZ-by-ASPH) or whether ASPH interacted with both diagnostic subgroup and FA variables (SZ-by-ASPH-by-FA) to predict outcome variables. Age, age^2 and sex were included as covariates in the models. For significant FA ROIs, RD and AD analyses were performed using the same models, as well.

© 2021 Wortinger LA et al. *JAMA Network Open.*

eAppendix 1. Supplementary Results

The *left* PLIC-FA showed a significant interaction ($F(2, 841) = 5.52$, $p = .004$) with the lowest FA in ASPH+ SZ patients (ASPH+ mean = 0.68, SD = 0.02; ASPH- mean = 0.69, SD = 0.02; $d = -0.47$) followed by ASPH+ BD patients (ASPH+ mean = 0.69, SD = 0.02; ASPH- mean = 0.69, SD = 0.02; $d = -0.28$), and a higher FA in ASPH+ HC (ASPH+ mean = 0.69, SD = 0.02; ASPH- mean = 0.69, SD = 0.02; $d = 0.26$) (eFigure 4A). The *right* PLIC-FA showed a significant interaction ($F(2, 841) = 5.03$, $p = .007$) with the lowest FA in ASPH+ SZ patients (ASPH+ mean = 0.67, SD = 0.02; ASPH- mean = 0.68, SD = 0.02; $d = -0.41$), followed by ASPH+ BD patients (ASPH+ mean = 0.68, SD = 0.02; ASPH- mean = 0.69, SD = 0.02; $d = -0.49$), and a higher FA in ASPH+ HC (ASPH+ mean = 0.69, SD = 0.02; ASPH- mean = 0.68, SD = 0.02; $d = 0.19$) (eFigure 4B).

RD analysis of PLIC revealed a significant interaction between ASPH and diagnostic subgroups ($F(2, 841) = 4.54$, $p = .01$). RD in the PLIC had the highest value in ASPH+ SZ patients (mean [SD]: ASPH+, 0.37 [0.02]; ASPH-, 0.37 [0.02]; $d = 0.37$) followed by ASPH+ BD patients (mean [SD]: ASPH+, 0.37 [0.02]; ASPH-, 0.36 [0.02]; $d = 0.42$), with similar effect sizes in both groups. In HC, lower RD was shown in ASPH+ group (mean [SD]: ASPH+, 0.36 [0.05]; ASPH-, 0.36 [0.02]; $d = -0.21$) (eFigure 5). This pattern was found in individual analyses of both the *left* and *right* PLIC-RD (eFigure 6).

The *left* PLIC-RD showed a significant interaction ($F(2, 841) = 4.23$, $p = .015$) with the highest RD in ASPH+ SZ patients (ASPH+ mean = 0.37, SD = 0.02; ASPH- mean = 0.36, SD = 0.02; $d = 0.37$) followed by ASPH+ BD patients (ASPH+ mean = 0.36, SD = 0.02; ASPH- mean = 0.35, SD = 0.02; $d = 0.26$), and a lower RD in ASPH+ HC (ASPH+ mean = 0.35, SD = 0.02; ASPH- mean = 0.36, SD = 0.02; $d = -0.25$) (eFigure 6A). The *right* PLIC-FA showed a significant interaction ($F(2, 841) = 4.12$, $p = .017$) with the highest RD in ASPH+ SZ patients (ASPH+ mean = 0.38, SD = 0.02; ASPH- mean = 0.37, SD = 0.02; $d = 0.33$) followed by ASPH+ BD patients (ASPH+ mean = 0.38, SD = 0.02; ASPH- mean = 0.37, SD = 0.02; $d = 0.55$), and a lower RD in ASPH+ HC (ASPH+ mean = 0.37, SD = 0.02; ASPH- mean = 0.37, SD = 0.02; $d = -0.14$) (eFigure 6B).

There were no significant associations between PLIC-FA, or RD, diagnostic subgroups and ASPH (neither main nor interaction effects) in predicting age of disease onset, CPZ, PANSS or GAF scores. Since groups differed significantly in Age, sensitivity analyses revealed that PLIC-FA still showed a significant effect of ASPH within the patient (F(1, 266) = 7.09, p = .008) and HC (F(1, 574) = 4.45, p = .04) groups, separately, covarying for age, age2 and sex. Pairwise comparisons revealed that PLIC-FA was significantly lower in patients with ASPH compared to those without (mean difference [SD]: -0.01 [0.07], $p = .008$) and significantly higher in HC with ASPH compared to those without (mean difference [SD]: 0.01 [0.07], $p = .04$).

© 2021 Wortinger LA et al. *JAMA Network Open.*

eTable. Twenty-five FA ROIs

					HC		PT	
ANCOVA	Main effect of Group	Main effect of ASPH	Group by ASPH interaction	ASPH	Mean	SD	Mean	SD
Projection fibers								
ACR	F (1, 843) = 8.86, p = .003	F (1, 843) = 0.72, p = .40	F (1, 843) = 0.43, p = .51	ASPH -	0.483	0.03	0.476	0.03
				ASPH +	0.482	0.03	0.472	0.03
ALIC	F (1, 843) = 6.60, p = .01	F (1, 843) = 1.48, p = .22	F (1, 843) = 1.35, p = .25	ASPH -	0.598	0.02	0.595	0.02
				ASPH +	0.598	0.02	0.589	0.02
CR	F (1, 843) = 8.11, p = .005	F (1, 843) = 1.20, p = .27	F (1, 843) = 3.44, p = .06	ASPH -	0.491	0.02	0.489	0.02
				ASPH +	0.493	0.02	0.482	0.02
CST	F (1, 843) = 0.49, p = .48	F (1, 843) = 0.25, p = .62	F (1, 843) = 2.22, p = .14	ASPH -	0.595	0.04	0.598	0.04
				ASPH +	0.599	0.04	0.591	0.04
IC	F (1, 843) = 4.17 p = .04	F (1, 843) = 1.61, p = .21	F (1, 843) = 2.97, p = .09	ASPH -	0.631	0.02	0.630	0.02
				ASPH +	0.632	0.02	0.623	0.02
PCR	F (1, 843) = 2.78, p = .10	F (1, 843) = 1.25, p = .26	F (1, 843) = 2.87, p = .09	ASPH -	0.489	0.03	0.489	0.03
				ASPH +	0.491	0.03	0.482	0.03
PLIC	F (1, 843) = 5.32, p = .02	F (1, 843) = 1.04, p = .31	F (1, 843) = 11.46, p = .001	ASPH -	0.684	0.02	0.687	0.02
				ASPH +	0.690	0.02	0.677	0.02
PTR	F (1, 843) = 6.13, p = .01	F (1, 843) = 0.56, p = .45	F (1, 843) = 0.30, p = .59	ASPH -	0.609	0.03	0.602	0.03
				ASPH +	0.608	0.03	0.598	0.03
RLIC	F (1, 843) = 0.35, p = .56	F (1, 843) = 1.08, p = .30	F (1, 843) = 0.04, p = .85	ASPH -	0.609	0.03	0.608	0.03
				ASPH +	0.607	0.03	0.604	0.03
SCR	F (1, 843) = 7.91, p = .005	F (1, 843) = 0.73, p = .39	F (1, 843) = 6.84, p = .009	ASPH -	0.501	0.02	0.500	0.02
				ASPH +	0.505	0.02	0.492	0.02
Association fibers								
CGC	F (1, 843) = 2.58, p = .11	F (1, 843) = 3.75, p = .05	F (1, 843) = 0.98, p = .32	ASPH -	0.606	0.04	0.603	0.04

© 2021 Wortinger LA et al. *JAMA Network Open.*

				ASPH +	0.602	0.04	0.592	0.04
CGH	F (1, 843) = 0.63, p = .43	F (1, 843) = 0.11, p = .74	F (1, 843) = 0.12, p = .73	ASPH -	0.522	0.05	0.528	0.05
				ASPH +	0.522	0.05	0.525	0.05
EC	F (1, 843) = 1.44, p = .23	F (1, 843) = 1.79, p = .18	F (1, 843) = 1.61, p = .21	ASPH -	0.457	0.03	0.457	0.03
				ASPH +	0.457	0.03	0.450	0.03
FXST	F (1, 843) = 0.03, p = .86	F (1, 843) = 0.77, p = .38	F (1, 843) = 0.03, p = .87	ASPH -	0.561	0.04	0.561	0.04
				ASPH +	0.559	0.04	0.558	0.04
IFO	F (1, 843) = 3.72, p = .05	F (1, 843) = 0.95, p = .33	F (1, 843) = 0.46, p = .50	ASPH -	0.473	0.02	0.470	0.02
				ASPH +	0.473	0.02	0.466	0.02
SFO	F (1, 843) = 0.87, p = .35	F (1, 843) = 2.70, p = .10	F (1, 843) = 0.05, p = .83	ASPH -	0.473	0.04	0.469	0.04
				ASPH +	0.466	0.04	0.463	0.04
SLF	F (1, 843) = 6.58, p = .01	F (1, 843) = 3.61, p = .06	F (1, 843) = 4.86, p = .03	ASPH -	0.532	0.03	0.530	0.03
				ASPH +	0.532	0.03	0.520	0.03
SS	F (1, 843) = 3.55, p = .06	F (1, 843) = 3.11, p = .08	F (1, 843) = 0.54, p = .46	ASPH -	0.569	0.03	0.565	0.03
				ASPH +	0.566	0.03	0.558	0.03
UNC	F (1, 843) = 1.26, p = .26	F (1, 843) = 4.32, p = .04	F (1, 843) = 1.22, p = .27	ASPH -	0.506	0.04	0.505	0.04
				ASPH +	0.502	0.04	0.492	0.04
Commissural fibers								
CC	F (1, 843) = 14.26, p < .001	F (1, 843) = 3.63, p = .06	F (1, 843) = 2.66, p = .10	ASPH -	0.737	0.03	0.730	0.03
				ASPH +	0.736	0.03	0.720	0.03
BCC	F (1, 843) = 10.22, p = .001	F (1, 843) = 3.49, p = .06	F (1, 843) = 1.50, p = .22	ASPH -	0.704	0.04	0.696	0.04
				ASPH +	0.702	0.04	0.683	0.04
GCC	F (1, 843) = 13.56, p < .001	F (1, 843) = 2.24, p = .14	F (1, 843) = 2.76, p = .10	ASPH -	0.719	0.03	0.712	0.03
				ASPH +	0.720	0.03	0.702	0.03
SCC	F (1, 843) = 11.00, p = .001	F (1, 843) = 3.01, p = .08	F (1, 843) = 2.48, p = .12	ASPH -	0.788	0.03	0.783	0.03
				ASPH +	0.787	0.03	0.774	0.03
FX	F (1, 843) = 5.75, p = .02	F (1, 843) = 4.26, p = .04	F (1, 843) = 0.17, p = .68	ASPH -	0.471	0.07	0.451	0.07
				ASPH +	0.453	0.07	0.439	0.07

© 2021 Wortinger LA et al. *JAMA Network Open.*

Average FA	F (1, 843) = 4.24, p = .04	F (1, 843) = 1.22, p = .27	F (1, 843) = 0.98, p = .32	ASPH -	0.464	0.02	0.461	0.02
				ASPH +	0.464	0.02	0.457	0.02

HC, healthy controls; PT, patients with bipolar disorder and schizophrenia; ACR, anterior corona radiata; ALIC, anterior limb of internal capsule; BCC, body of corpus callosum; CC, corpus callosum; CGC, cingulum; CGH, cingulum (hippocampal portion); CR, corona radiata; CST, corticospinal tract; EC, external capsule; FA, fractional anisotropy; FX, fornix; FXST, fornix stria terminalis; GCC, genu of corpus callosum; IC, internal capsule; IFO, inferior fronto occipital fasciculus; PCR, posterior corona radiata; PLIC, posterior limb of internal capsule; PTR, posterior thalamic radiation; RLIC, retrolenticular part of IC; ROI, region of interest; SCC, splenium of corpus callosum; SCR, superior corona radiata; SFO, superior fronto-occipital fasciculus; SLF, superior longitudinal fasciculus; SS, sagittal stratum; UNC, uncinate.

© 2021 Wortinger LA et al. *JAMA Network Open.*

eFigure 1. FA Comparison Before and After ComBat Harmonization for Scanners

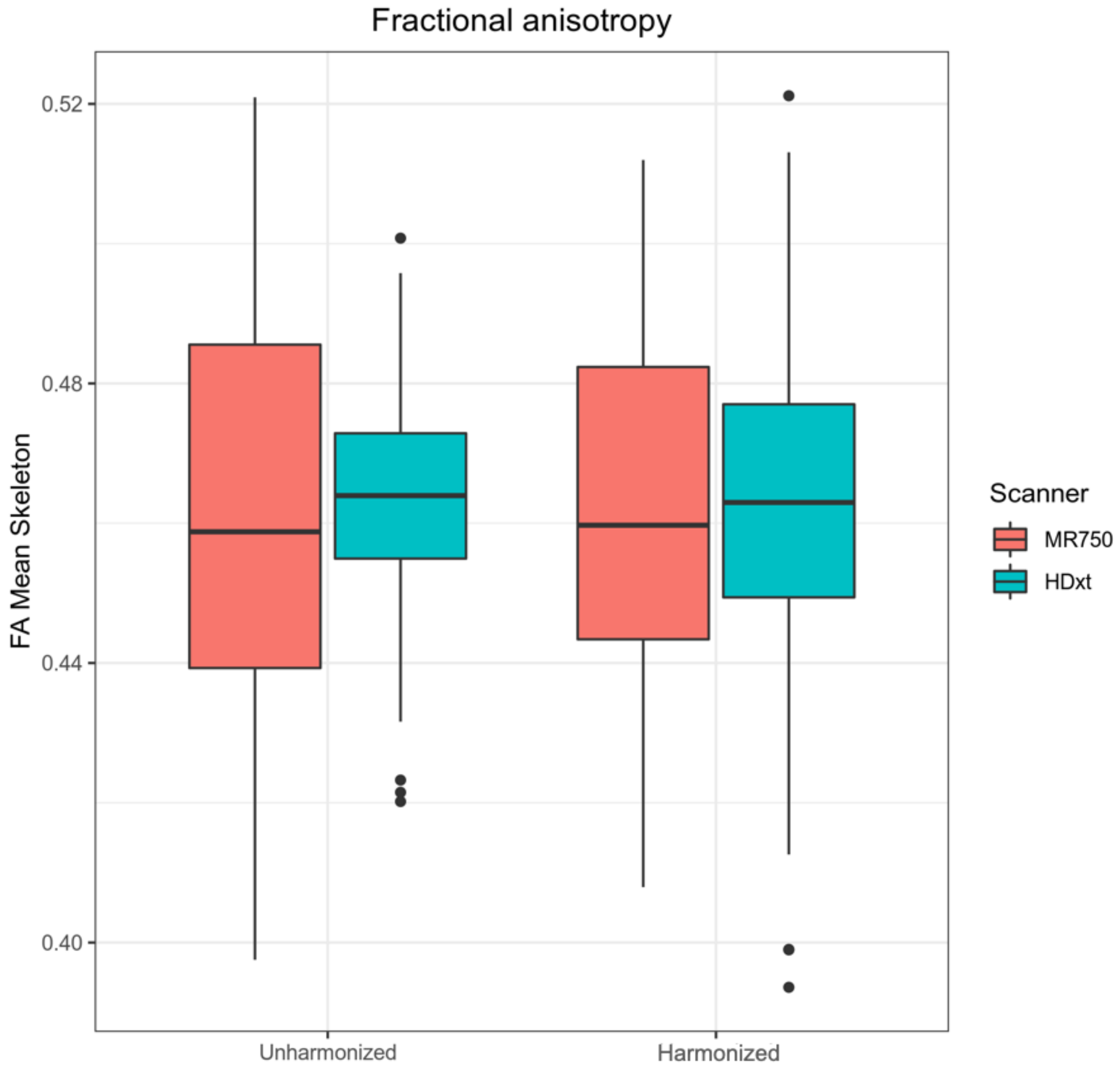

Figure 1. Fractional anisotropy (FA) comparison before and after ComBat harmonization for scanners.

© 2021 Wortinger LA et al. *JAMA Network Open.*

eFigure 2. RD Comparison Before and After ComBat Harmonization for Scanners

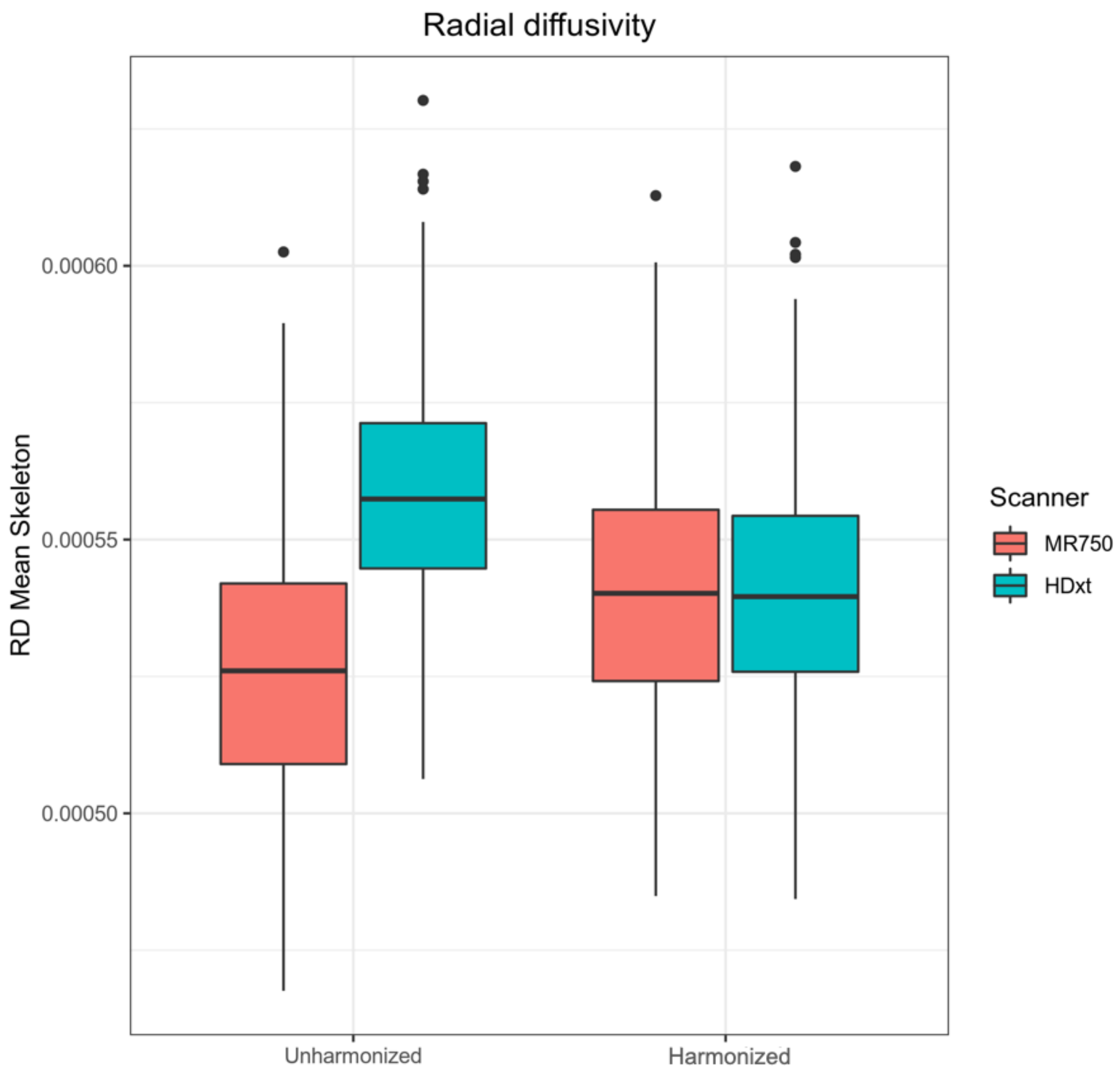

Figure 2. RD comparison before and after ComBat harmonization for scanners.

© 2021 Wortinger LA et al. *JAMA Network Open.*

eFigure 3. AD Comparison Before and After ComBat Harmonization for Scanners

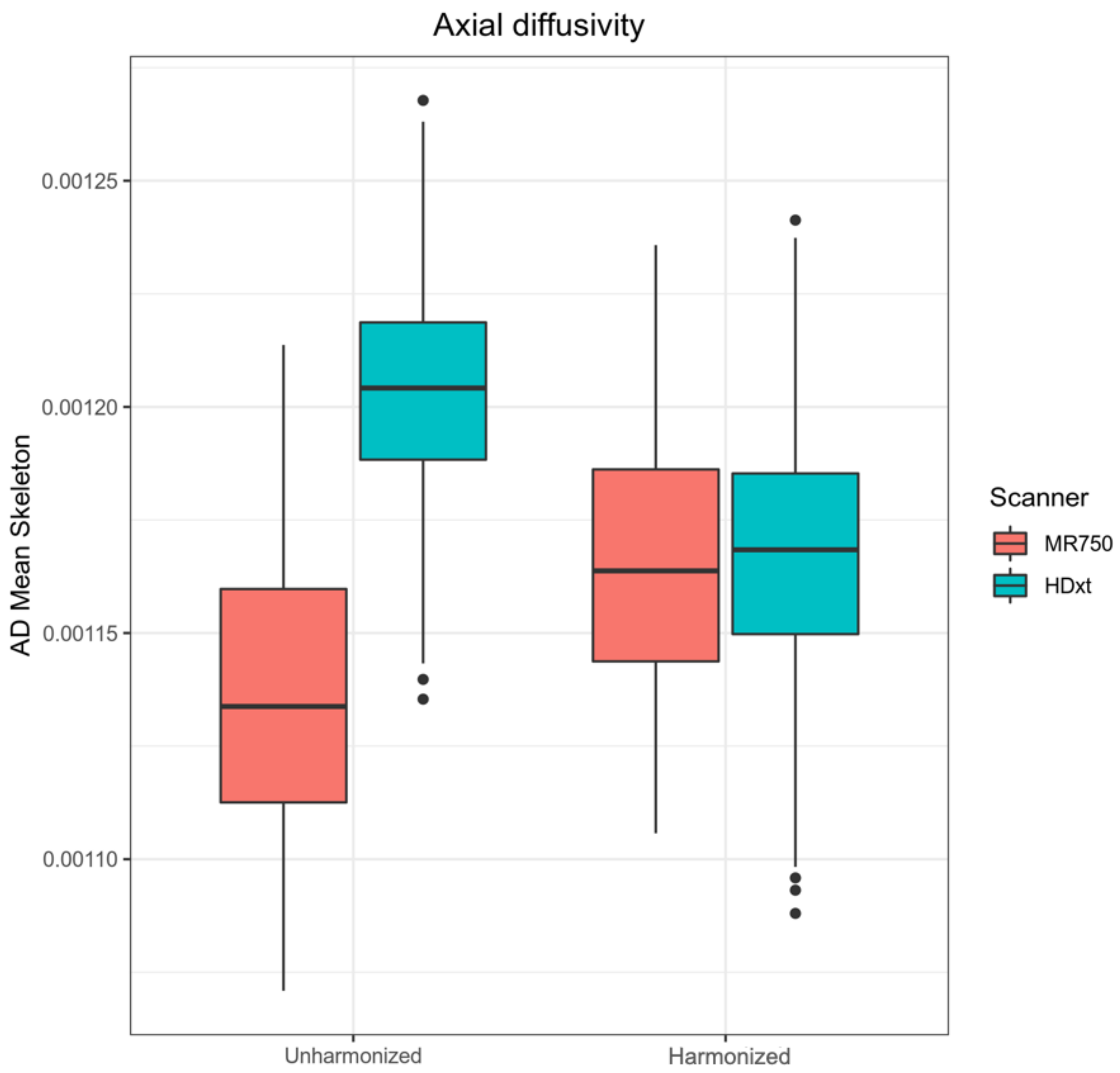

Figure 3. Axial diffusivity (A) comparison before and after ComBat harmonization for scanners.

© 2021 Wortinger LA et al. *JAMA Network Open.*

eFigure 4. Diagnostic Subgroup × Birth Asphyxia Interaction for Fractional Anisotropy in the Left and Right Hemisphere Posterior Limb of the Internal Capsule

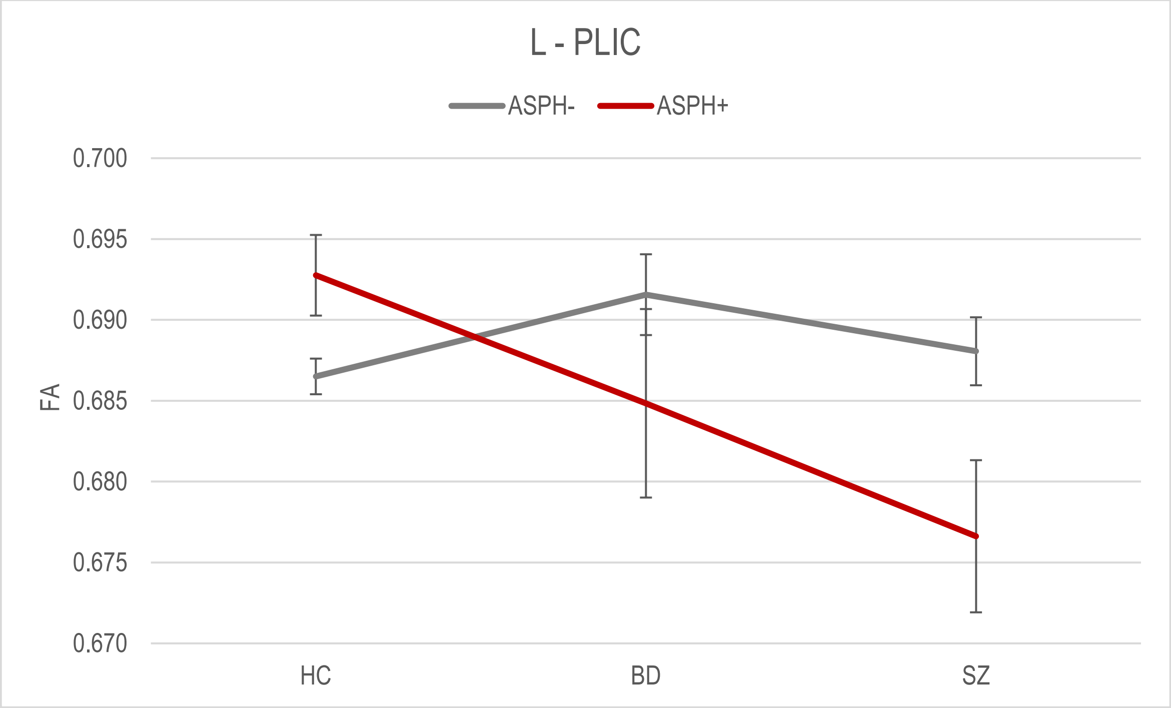

A

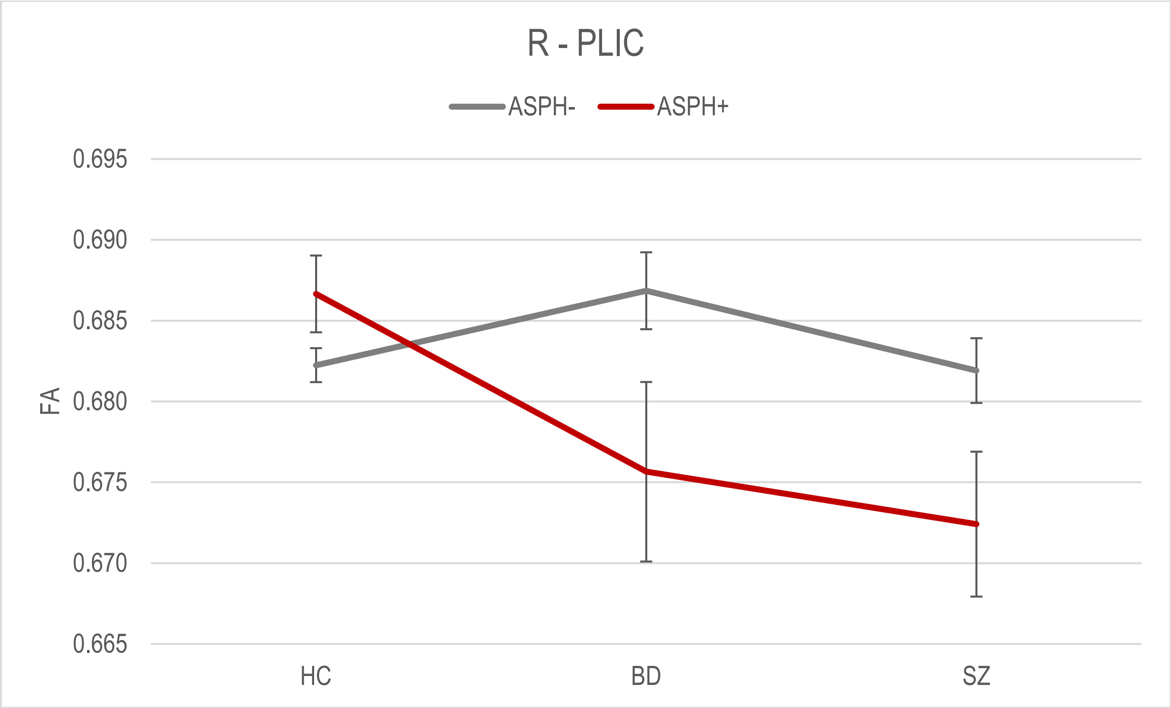

B

Figure 4. A significant diagnostic subgroup-by-birth asphyxia (ASPH) interaction was found for fractional anisotropy (FA) in both the left (L; panel A) and right (R; panel B) hemisphere posterior limb of the internal capsule (PLIC) revealing lower FA in both patients with schizophrenia (SZ) and bipolar disorder (BD) who experienced ASPH (+) compared to those who did not (-), a difference not observed in healthy controls (HC). Error bars represent standard error of the mean.

© 2021 Wortinger LA et al. *JAMA Network Open.*

eFigure 5. Diagnostic Subgroup × Birth Asphyxia Interaction for Radial Diffusivity in the Posterior Limb of the Internal Capsule

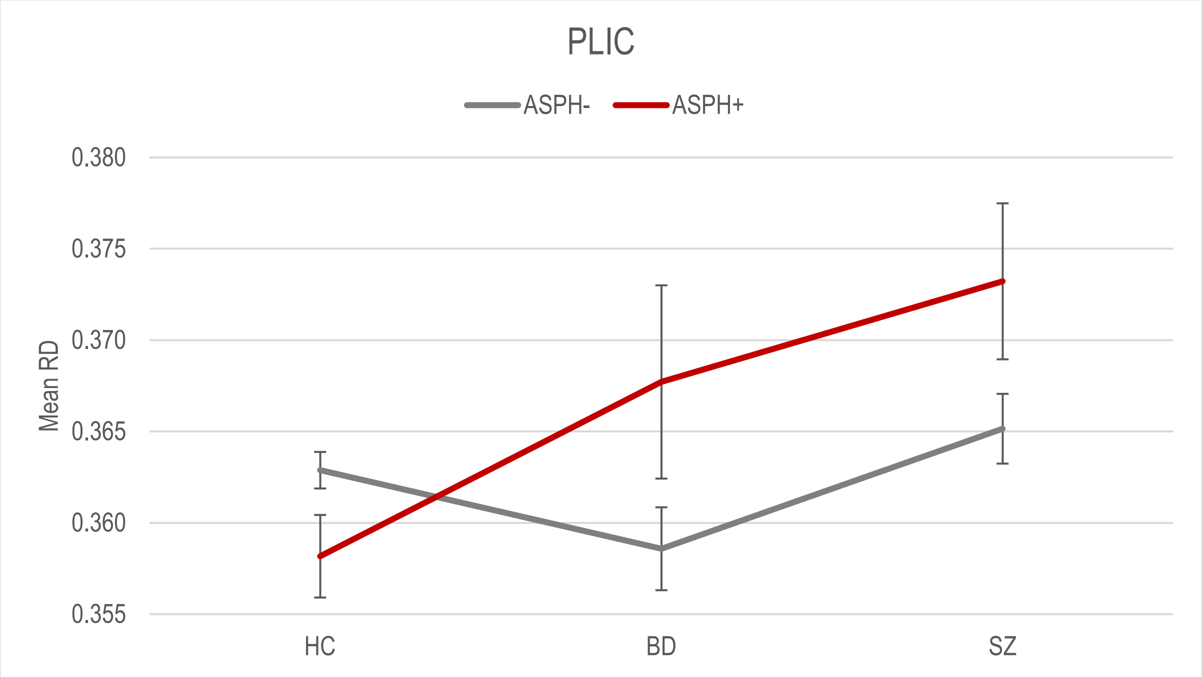

Figure 5. A significant diagnostic subgroup-by-birth asphyxia (ASPH) interaction was found for radial diffusivity (RD) in the posterior limb of the internal capsule (PLIC) revealing higher RD in both patients with schizophrenia (SZ) and bipolar disorder (BD) who experienced ASPH (+) compared to those who did not (-), a difference not observed in healthy controls (HC). Error bars represent standard error of the mean.

© 2021 Wortinger LA et al. *JAMA Network Open.*

eFigure 6. Diagnostic Subgroup × Birth Asphyxia Interaction for Radial Diffusivity in the Left and Right Hemisphere Posterior Limb of the Internal Capsule

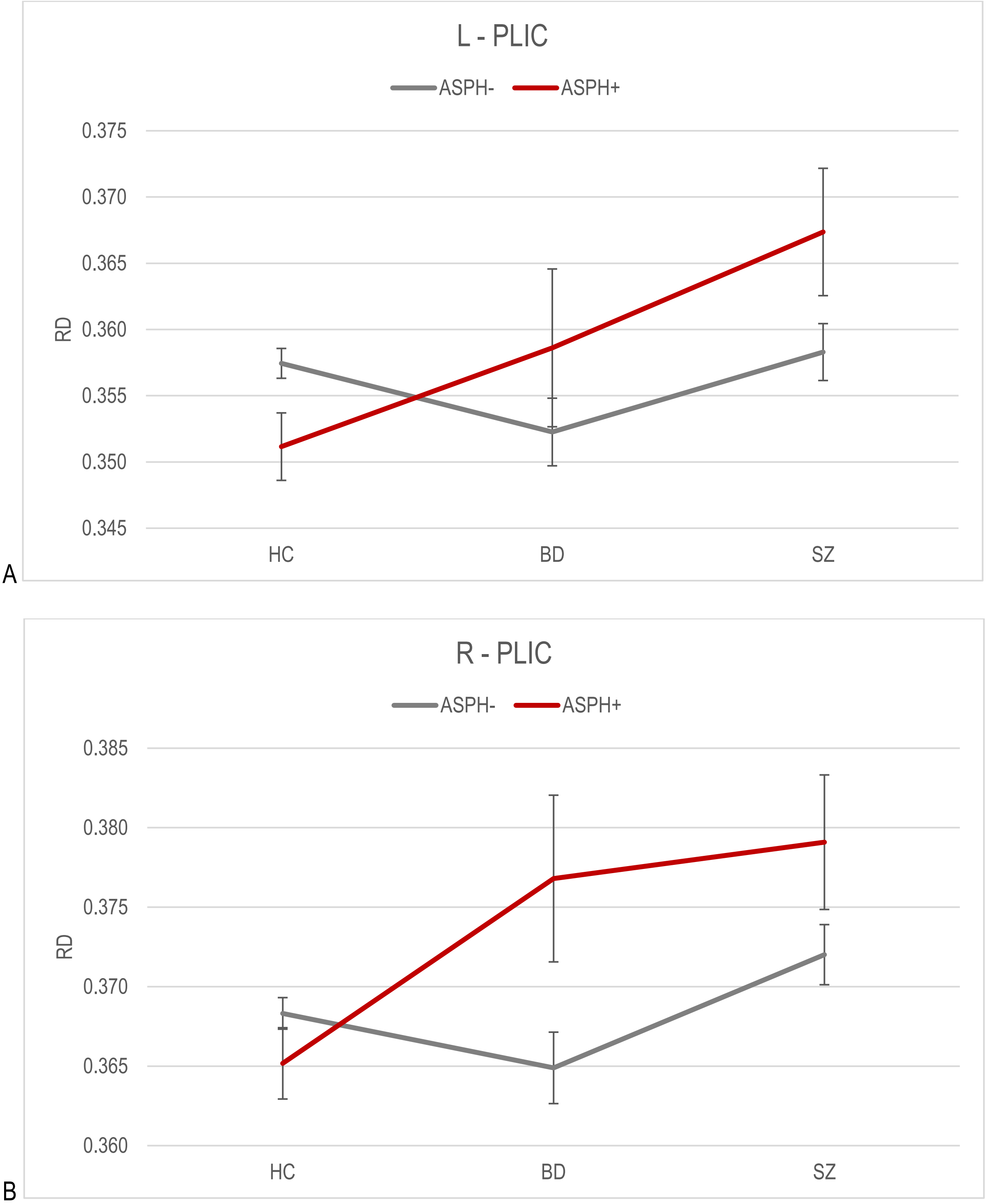

Figure 6. A significant diagnostic subgroup-by-birth asphyxia (ASPH) interaction was found for radial diffusivity (RD) in both the left (L; panel A) and right (R; panel B) hemisphere posterior limb of the internal capsule (PLIC) revealing higher RD in both patients with schizophrenia (SZ) and bipolar disorder (BD) who experienced ASPH (+) compared to those who did not (-), a difference not observed in healthy controls (HC). Error bars represent standard error of the mean.

© 2021 Wortinger LA et al. *JAMA Network Open.*

eAppendix 2. TOP MRI Grading Scheme

The MRI images are graded from 1 to 4, with the following criteria:

1. No brain pathology observed (including sinus densities).
2. Minor changes, which may be included in normal variation, that do not require further clinical examination. For example:
    - ventricular asymmetry
    - increased amount of CSF
    - cortical and cerebellar atrophy
    - septum pellucidum changes
    - non-specific changes in subcortical white matter.
    - smaller cysts (<1 x 1 cm)
    - minor pituitary changes

3. Changes that are clearly not of a normal variation and require further clinical examination. For example:
    - cysts (> 1 x 1cm)
    - empty sella
    - widespread / major pituitary changes
    - Arteriovenous (AV) malformations

4. Clear / more extensive pathology that requires further clinical examination (Multiple sclerosis (MS), tumor, infarction).

1 and 2 are included, and 3 and 4 are excluded in clinical MRI studies in TOP.

© 2021 Wortinger LA et al. *JAMA Network Open.*

eReferences.

1. Spitzer RL, Williams JB, Gibbon M, First MB. Structured clinical interview for DSM-III-R-patient version (SCID-P). *New York, NY: Biometrics Research Department, New York State Psychiatric Institute.* 1988.
2. Pedersen G, Hagtvet KA, Karterud S. Generalizability studies of the Global Assessment of Functioning-Split version. *Compr Psychiatry.* 2007;48(1):88-94.
3. Kay SR, Fiszbein A, Opler LA. The positive and negative syndrome scale (PANSS) for schizophrenia. *Schizophr Bull.* 1987;13(2):261-276.
4. Spitzer RL, Williams JB, Kroenke K, et al. Utility of a new procedure for diagnosing mental disorders in primary care. The PRIME-MD 1000 study. *Jama.* 1994;272(22):1749-1756.
5. Katzman GL, Dagher AP, Patronas NJ. Incidental findings on brain magnetic resonance imaging from 1000 asymptomatic volunteers. *Jama.* 1999;282(1):36-39.
6. Sommer IE, de Kort GA, Meijering AL, et al. How frequent are radiological abnormalities in patients with psychosis? A review of 1379 MRI scans. *Schizophr Bull.* 2013;39(4):815-819.

© 2021 Wortinger LA et al. *JAMA Network Open.*
